# A nanocomposite hydrogel delivery system for mesenchymal stromal cell secretome

**DOI:** 10.1186/s13287-020-01712-9

**Published:** 2020-05-27

**Authors:** K. Shoma Suresh, Samatha Bhat, Bharath Raja Guru, Manjunatha S. Muttigi, Raviraja N. Seetharam

**Affiliations:** 1 Stempeutics Research Private Limited, Shirdi Sai Baba Cancer Hospital, Manipal, 576104 India; 2 Department of Biotechnology, Manipal Institute of Technology, Manipal Academy of Higher Education, Manipal, 576104 India; 3 Manipal Academy of Higher Education, Manipal, 576104 India

**Keywords:** Mesenchymal stromal cells, Conditioned medium, Nanocomposite hydrogel, Secretome, Delivery model

## Abstract

**Background:**

Mesenchymal stromal cell conditioned medium (MSC-CM) contains a cocktail of bioactive factors that act synergistically to induce therapeutic effects. This has been clearly demonstrated by in vivo applications of MSC-CM, but the establishment of controlled delivery systems is an unmet requirement for clinical translation.

**Methods:**

We developed a nanocomposite-hydrogel (NP-H) comprised of poly-L-lactide nanoparticles (NPs) embedded in gelatin/hyaluronic acid (Gel/HA) hydrogel as a delivery vehicle for MSC-CM. First, we optimized the culture conditions for bone marrow-derived MSCs using serum-containing medium (SCM) and serum-free medium (SFM) and characterized the corresponding CM (serum-containing conditioned medium (ScCM) and serum-free conditioned medium (SfCM), respectively) for its potency and xeno markers. Then we prepared a composite matrix followed by physiochemical characterization and functional assays were performed.

**Results:**

Nanocomposite hydrogel displayed an even distribution of NPs along with high porosity (> 60%) and swelling ratios > 1500%, while its protein release pattern corresponded to a mix of degradation and diffusion kinetics. Functional evaluation of the composites was determined using MSCs and human fibroblasts (HFFs). The cells seeded directly onto the composites displayed increasing metabolic activities over time, with ScCM-NP-H groups having maximum activity. The cells treated in vitro with 5% and 10% extracts of ScCM-NP-H and SfCM-NP-H exhibited a dose- and duration-dependent response. Cell activities reduced considerably for all groups, except 10% ScCM-NP-H, which displayed a significant increase over time.

**Conclusion:**

We observed that sustained release of MSC-CM is required to prevent dose-dependent cytotoxicity. The proposed nanocomposite hydrogel for MSC-CM delivery can open up a new array for its clinical application.

## Background

Mesenchymal stromal cells (MSCs) consist of a heterogeneous population of spindle-shaped cells derived from the stroma of various adult tissues. They are non-hematopoietic and capable of self-renewal and have the capacity to differentiate into mesoderm lineages under in vitro conditions [1–3]. The International Society for Cellular Therapy (ISCT) has proposed three criteria to define MSCs: (a) they should be adherent under standard culture conditions; (b) exhibit a specific set of surface antigens—CD105, CD73, and CD90 (≥ 95%) and CD45, CD34, CD11b, CD19 and HLA Class II (≤ 2%); and (c) differentiate into osteoblasts, chondrocytes, and adipocytes under standard in vitro conditions [4]. MSCs have garnered special attention in the fields of tissue engineering and regenerative medicine due to their potential for tissue repair and regeneration, their immunomodulatory capabilities, and their low immunogenicity in comparison to other stem cells [5, 6].

MSCs mediate cellular processes mainly by secreting large number of bioactive molecules, which include growth factors, cytokines, chemokines, hormones, and extracellular vesicles [7, 8]. These complex set of molecules are collectively termed as the MSC “secretome” and the culture medium into which they are secreted is referred to as the “MSC conditioned medium” (MSC-CM). MSC-CM is known to exhibit therapeutic properties [9] and can be used as a substitute for the cells, without hindering the efficacy of the therapeutic effect when treating pathological conditions [10, 11]. The secretome can directly mediate cell to cell communication or induce neighboring cells to secrete bioactive factors [12].

Secretome therapies are allogeneic and can be prepared in advance as an “off the shelf” therapy for various conditions, similar to vaccines and monoclonal antibodies. Also, freeze-drying or lyophilization of CM allows for easy storage and transportation when compared to cell-based products, which require cryopreservation and cold chain management [12, 13]. MSC-CM has shown therapeutic effects in animal models of numerous pathological conditions such as myocardial infarction, stroke, tissue ischemia, spinal cord injury, wound healing, arthritis, Parkinson’s disease, musculoskeletal diseases, acute lung injuries, liver diseases, and kidney diseases [7, 14–17].

MSC-CM has a complex composition that comprises secreted proteins and other bioactive factors present in the culture medium. The use of bovine serum in culture medium limits translational benefits due to xenogeneic adverse effects [18]. Thus it would be beneficial to develop clinical-grade MSC-CM that is free of any serum-based or xenogeneic components. This can be done by culturing the MSCs under reduced serum conditions and later switching the cultures to serum-free medium [19–21]. Many commercially available xeno-free/serum-free media have been shown to support MSC expansion and growth, but it is necessary to optimize culture conditions in order to obtain a specific product for clinical application [20, 21].

Nanocomposite hydrogels (NP-H) comprising polymer nanoparticles (NPs) incorporated within a hydrogel matrix have been effective for drug delivery applications [22, 23] and thus can be applied for encapsulation of MSC-CM. Poly-lactic acid (PLA) is a biodegradable thermoplastic polymer commonly used for the preparation of NPs and is degraded by hydrolysis to produce monomeric units of lactic acid, which can be easily eliminated from the body [24]. A hydrogel (H) is a 3D network of crosslinked polymers that have been used for drug delivery and tissue engineering applications [22]. Gelatin (Gel) and hyaluronic acid (HA) are commonly utilized in hydrogel synthesis and have shown excellent biocompatibility. The synergistic effect of these biopolymers has an added advantage, allowing for increased swelling of the hydrogels along with added hydrolytic stability [25–28].

In this study, we hypothesized that the preparation of MSC-CM and encapsulation into an NP-H composite delivery vehicle for controlled delivery can open up a new array of MSC-CM clinical applications. In this regard, we optimized MSC culture conditions in serum-containing (SCM) and serum-free medium (SFM) and characterized MSC-CM for its potency markers. The CM was lyophilized and encapsulated into PLA-NPs and physical characterization was carried out. The CM-NPs were further embedded into Gel/HA hydrogels to form NP-H composites, which were characterized for morphological and physiochemical parameters, followed by a functional evaluation using MSCs and HFF under MTT assay conditions. In brief, we have observed that the controlled release of MSC-CM prevents dose-dependent toxicity to the cells in vitro.

## Materials and methods

### Materials

KnockOut™ Dulbecco’s modified Eagle’s medium (1×; DMEM-KO™), Glutamax™ (100×), trypsin-ethylene diamine tetra acetic acid (EDTA; 1 mM), and streptomycin (1×) were purchased from ThermoFisher Scientific, USA. Fetal bovine serum (heat-inactivated FBS; Australia origin) was obtained from Hyclone, USA. Dulbecco’s phosphate buffer saline (DPBS; 1×) was obtained from GIBCO, USA. Gelatin (type A) from porcine skin with gel strength 300 g bloom, basic fibroblast growth factor (bFGF), D-(+)-trehalose dihydrate, and Bradford reagent were procured from Sigma Aldrich, USA. MSC NutriStem® XF Basal Medium and MSC NutriStem® XF Supplemental Mix were acquired from Biological Industries, USA. The BSA immunoassay kit was purchased from R and D systems, USA, and Poly (DL-lactide) (PLA) acid terminated with inherent viscosity 0.16–0.25 dL/g was from Durect Corporation, USA. Hyaluronic acid (HA) with Mw = 100–150 kDa (medical grade) was obtained from Xian Natural Field Bio-Technique Co. Ltd., China; N-hydroxy succinimide (NHS) and 1-ethyl-3-(3-dimethylaminopropyl)-carbodiimide hydrochloride (EDC-HCl) extra pure were purchased from Spectrochem Pvt. Ltd., India and SRL, India, respectively. Bone marrow-derived MSCs (BM-MSCs) were initially isolated from the bone marrow aspirates of the iliac crest of human donors after signed consent and following institutional ethical guidelines. Human fibroblasts (HFF-1) are procured from ATCC and used for functional assay.

### MSC culture

MSCs from multiple donors were pooled and cultured until passage 3 (P3) in serum-containing media [20]. Cryopreserved BM-MSCs in P3 were thawed, revived, and subsequently used for all experiments. MSCs were cultured under two conditions: (a) SCM (DMEM-KO™ supplemented with 10% FBS, 1× Glutamax™, 1% streptomycin, and 2 ng/mL bFGF) and (b) SFM (NutriStem® XF basal medium containing NutriStem® Supplemental solution and 1% streptomycin). For SFM, the culture vessels were pre-coated with MSC attachment solution. P3 MSCs were seeded in T-75 flasks at a density of 1000 cells/cm^2^ in 15 mL media and incubated at 37 °C under 5% CO_2_ and 95% relative humidity. Medium changes for the cultures performed at 50% confluency. Once the cells reached 80–90% confluency, they were washed twice with DPBS and harvested using trypsin-EDTA. Then the cells were seeded in one Cell-STACK (one-CS) (Corning, USA) at a density of 1000 cells/cm^2^ in 150 mL complete media. A complete medium change was carried out at 50 to 60% confluency (on culture day 6) for both the cultures. At 80% confluency (on culture day 8), ScCM and SfCM were collected from one-CS and centrifuged to remove any cell debris. Culture medium collected on day 8 from SCM and SFM cultures as ScCM and SfCM, respectively, were used for study. Extracellular vesicles (EVs) were not removed from the CM collected from both SCM and SFM cultures. The MSCs were then harvested using trypsin-EDTA and characterized for cell count, viability, average diameter, and circularity using the automated Vi-CELL XR 2.03 (Beckman Coulter, Inc.). The values were calculated as the average result of 50 image analyses in Vi-CELL. MSC-CM was used for immediate characterization or stored at − 80 °C until required.

### Analysis of surface marker expression

The expression of surface markers of MSCs cultured using SCM and SFM at passage 5 was evaluated by flow cytometry [20]. Briefly, cell suspensions were incubated with fluorescein isothiocyanate (FITC) or phycoerythrin (PE)-conjugated antibodies such as CD34-PE, CD90-PE, CD105-PE (Chemicon), CD45-FITC, CD73-PE, and HLA-DR-FITC (BD Pharmingen, USA) in the dark for 30 min at room temperature. Appropriate isotype controls were used to analyze antigenic events. Flow cytometry was performed using an EasyCyte and analyzed using Guava Express Pro software (Guava Technologies, USA).

### Differentiation assay

Trilineage differentiation for P5 BM-MSCs cultured in SCM and SFM was performed simultaneously, as per the manufacturer’s instructions. Briefly, osteogenic differentiation was induced using an osteogenic differentiation medium (Stempro™ ostogenic differentiation kit). Differentiation was assessed by determining the calcium deposition from the cells, using von Kossa’s staining. Adipogenic differentiation was induced by means of an adipogenic differentiation medium (Stempro™ adipogenic differentiation kit). The differentiation was assessed by Oil Red O staining. Chondrogenic differentiation was stimulated by using a chondrogenic differention medium (Stempro™ chondrogenesis differentiation kit) in non-adherent concave 96-well plates. Safranin-O stain was used to evaluate the extent of chondrogenic differentiation. The lineage differentiation images were captured using a Nikon Eclipse 90i microscope (Nikon, Japan; www.nikon.com) and Image-Pro Express software (Media Cybernetics, Silver Springs, MD, USA; www.mediacy.com).

### MSC-CM

ScCM and SfCM collected from P5 MSC cultures were characterized for total protein content and the presence of potency markers VEGF, TGF-β, and xeno marker BSA using ELISA. This was followed by concentrating and characterizing lyophilized CM.

#### MSC-CM characterization

##### Protein quantification

The total protein content in ScCM and SfCM was quantified using the Bradford assay with BSA standards.

##### TGF-β1 and VEGF immunoassay

The levels of TGF-β1 and VEGF in ScCM and SfCM were analyzed using a quantitative sandwich enzyme immunoassay technique. The assay was conducted according to the Quantikine® Human TGF-β1 and VEGF Immunoassay kit-based protocol (R and D systems, USA). Briefly, standards, controls, and diluted CM were pipetted into microplates pre-coated with respective monoclonal antibodies (mAbs). After a washing step, enzyme-linked polyclonal antibody specific for human TGF-β1 or VEGF was added to the wells. Unbound reagents were washed and a substrate solution was added, which develops color proportional to the amount of VEGF/TGF-β1 present. The color development was stopped and optical density was measured at 450 nm using a Versa Max microplate reader (Molecular Devices, USA). The samples were assayed in duplicates and the amount of VEGF and TGF-β1 secreted was represented as nanograms/milliliter/million cells.

##### BSA immunoassay

The level of bovine serum albumin (BSA) in SfCM was measured using a sandwich enzyme immunoassay technique according to the kit-based protocol.

#### Concentration of MSC-CM

ScCM and SfCM were concentrated by lyophilization using 4% trehalose as a cryoprotectant [29]. The CM was frozen at − 80 °C overnight and then lyophilized under vacuum at − 110 °C under pressure of < 1 mbar for 72 h. The CM was stored at − 20 °C until required. The protein content of the lyophilized CM (L-CM) was analyzed using the Bradford assay.

### Nanoparticle preparation and MSC-CM encapsulation

Nanoparticles (NPs) were prepared using the double emulsion solvent evaporation method with some modifications from those presented in the literature [30]. The NP groups were denoted as ScCM-NPs and SfCM-NPs. First, the lyophilized CM (L-ScCM or L-SfCM) was dissolved in water to form the aqueous phase (w1) and PLA was dissolved in dichloromethane to form the oil phase (o). Next, (w1) was added to (o) such that the ratio of L-CM: polymer was 1:2. The mixture was sonicated for 1.5 min in an ice bath, followed by addition of 2% PVA solution and sonication for 4.5 min to form a (w1/o/w2) emulsion. The emulsion magnetically stirred overnight and then the NP solution was washed thrice with water by centrifugation for 30 min each time at 10,000 rpm at 4 °C. The NPs were resuspended in 1 mL water and used for hydrogel synthesis. For characterization studies, the NP solution was frozen at − 80 °C overnight and lyophilized at − 110 °C for 48 h. The lyophilized NPs were stored at − 20 °C.

#### Nanoparticle characterization

##### Particle size and zeta potential

Dynamic light scattering (DLS) was used to determine the particle size and zeta potential of the NPs. For this, 1 mg of the lyophilized NPs was resuspended in water and serially diluted. The NPs were evenly dispersed in solution for 30 s in an ultrasonic water bath and analyzed using DLS (MalvernZetaSizer; Malvern panalytical, UK).

##### Protein loading capacity

The protein loading was evaluated by the NP digestion method. For this, 1 mg of lyophilized NPs was dissolved by adding a 1-mL solution of 100 mM sodium hydroxide (NaOH) and 5% SDS. The solution was incubated with continuous agitation at 37 °C for 1 h and then centrifuged at 12,000×*g* for 10 min. The supernatant was collected and analyzed for protein content using the Bradford assay. All the studies were carried out in triplicate. The loading capacity of NPs was calculated using the following formula [31]:

$$ \mathrm{Protein}\ \mathrm{loading}\ \left(\%\right)=\mathrm{Quantified}\ \mathrm{protein}/\mathrm{weight}\ \mathrm{of}\ \mathrm{NP}\times 100. $$

##### Protein release study

For this, 1 mg of the NPs was dispersed in 1 mL of DPBS and placed in a shaker incubator at 37 °C. At fixed time intervals, the samples were centrifuged at 1200×*g* for 10 min and 100 μL of the supernatants was collected. Next, 100 μL of fresh DPBS was added to the vials to maintain the overall volume of the release medium. The amount of protein released was determined by the Bradford assay. All studies were carried out in triplicate.

### NP-H composite preparation

#### Hydrogel preparation and NP embedding

The hydrogels were prepared using a method formulated by Yang et al. [32] with modifications. Gelatin (3% w/v) was dissolved in water at 50 °C and HA (3% w/v) solution was added at a ratio of Gel: HA = 4:1. EDC and NHS stock solutions (in MilliQ water) were added to the Gel/HA polymer solution to achieve final crosslinker concentrations as 25 mM and 10 mM, respectively. The hydrogel solution was crosslinked at 37 °C for 2 h and solidified Gel/HA hydrogel was washed with water to remove the residual reagents. For NP-H composite synthesis, ScCM-NPs or SfCM-NPs were added at a concentration of 3 mg/mL of the hydrogel solution, just before initiation of the crosslinking step. Then, EDC/NHS was added and the same protocol was followed for gelation. The final nanocomposite groups were (a) Gel/HA hydrogel (H), b) Gel/HA hydrogel embedded with ScCM-NP (ScCM-NP-H), and (c) Gel/HA hydrogel embedded with SfCM-NP (SfCM-NP-H). Samples for the protein release study and cell culture-based functional assays were used as such, while the samples for physiochemical characterization were frozen overnight at − 20 °C and lyophilized for 48 h.

#### NP-H composite characterization

##### FTIR analysis

Chemical characterization of H was carried out by Fourier transform infrared (FTIR) spectroscopy using JASCO FT/IR-6300 typeA spectrometer. The lyophilized H, Gel, and HA were mixed with KBr and pressed to form pellets. The spectra were recorded in the range of 4000 to 400 cm^−1^ at a resolution of 2 cm^−1^.

##### Morphology by SEM

The cross-sectional morphology of the H and NP-H groups was assessed using EVO MA18 scanning electron microscopy (SEM) and Jeol JSM 6380LA analytical SEM. A thin cross-section of the sample was placed on double-sided adhesive carbon tape mounted on a metallic stub. The samples were sputter-coated with gold for 200 s and viewed under SEM.

##### Swelling ratio and degradation rate

Here, 10 mg of lyophilized H and NP-H samples (*W*_*d*_) were immersed in 1 mL DPBS at room temperature. After 1 h and 24 h, the swollen samples were drained of excess water and weighed again (*W*_*s*_). The swelling ratios were calculated as follows:

$$ \mathrm{Swelling}\ \mathrm{ratio}\ \left(\mathrm{SR}\right)=\left({W}_s-{W}_d\right)/{W}_d\times 100. $$

For analysis of the degradation rate, the lyophilized samples were weighed (*W*_0_) and incubated in 1 mL of DPBS at 37 °C. At specific time points (hour 6, days 1, 3, 5, and 7), samples were removed from water, lyophilized, and weighed again (*W*_*t*_). The degradation rate was calculated as follows [33]:

$$ \mathrm{Degradation}\ \mathrm{rate}\ \left(\mathrm{DR}\right)=\left({W}_0-{W}_t\right)/{W}_0\times 100 $$

##### Porosity measurement

Porosity was determined indirectly by the ethanol displacement method [32]. Briefly, the pre-weighed lyophilized samples were immersed for 5 min in a known volume of ethanol (V1) and placed in vacuum until air bubble formation stopped. The total volume of the sample and liquid was measured (V2). Then the samples were removed from the ethanol and the remaining volume was measured (V3). The porosity of the samples was calculated using the formula:

$$ \mathrm{Porosity}\ \left(\varepsilon \right)=\left(\mathrm{V}1-\mathrm{V}3\right)/\left(\mathrm{V}2-\mathrm{V}3\right) $$

##### Protein release study

NP-H composites were weighed, immersed in DPBS, and placed in a shaker incubator at 37 °C. At 1 h and 6 h and 1-, 2-, 5-, and 8-day time points, 100 μL of the supernatant was collected and substituted with an equal volume of fresh DPBS. The protein released from the composites was quantified using the Bradford assay. All studies were carried out in triplicates.

### Functional evaluation of NP-H composites

#### Cell seeding on hydrogels

Functional evaluation of the H and NP-H composites was carried out using MSCs and human fibroblasts (HFF). The H and NP-H composites were added to 48-well culture plates that were washed twice with DPBS. The hydrogels were soaked in 200 μL of DMEM-KO for 24 h. The saturated hydrogels were seeded with 2 × 10^5^ cells (MSCs or HFFs) and incubated in a CO_2_ incubator. The groups were as follows: (a) MSCs in H, (b) MSCs in ScCM-NP-H, (c) MSCs in SfCM-NP-H, (d) MSCs 2-D control (cells cultured without hydrogels), (e) HFF in H, (f) HFF in ScCM-NP-H, (g) HFF in SfCM-NP-H, and (h) HFF 2-D control (cells cultured without hydrogels).

Metabolic activity of the cells was assessed using an MTT assay. The yellow MTT (3-(4, 5-dimethylthiazolyl-2)-2, 5-diphenyltetrazolium bromide) is reduced to purple-colored insoluble formazan crystals in metabolically active cells. After 24 and 48 h, 25 μL of MTT reagent (5 mg/mL in DPBS) was added to each well and incubated for 2 h till the formation of a visible purple precipitate. Then, 100 μL of DMSO was added to each well and left for 10–15 min to solubilize the formazan crystals. The absorbance was read at 570 nm with a reference wavelength of 620 nm using a SpectraMax M3 microplate reader.

The metabolic activity of the cells was expressed in terms of relative cell viability (%) normalized with respect to the 2D control, using the following formula:

$$ \mathrm{Relative}\ \mathrm{cell}\ \mathrm{viability}\ \left(\%\right)=\left(\mathrm{OD}\ \mathrm{of}\ \mathrm{sample}/\mathrm{OD}\ \mathrm{of}\ 2\mathrm{D}\ \mathrm{control}\right)\times 100 $$

#### Cell treatment with hydrogel extracts

Here, 1 mL each of the H and NP-H groups was extracted in DMEM-KO for 48 h by incubation at 37 °C. Supernatants were separated by centrifugation at 12,000×*g* for 10 min and filtered through 0.22 μm syringe filters. These extracts were used for cell treatments.

MSCs/HFFs in culture medium containing 10% FBS with 1% glutamax were seeded into 48-well plates at a density of 20,000 cells per well. The plates were incubated at 37 °C with 5% CO_2_ and 95% relative humidity to allow cellular attachment. After 24 h, the media was carefully aspirated from the wells and 300 μL of the diluted extracts in DMEM-KO were added. The experimental groups were as follows: (a) H 5% extract (G1), (b) H 10% extract (G2), (c) SCM-CM-NP-H 5% extract (G3), (d) SCM-CM-NP-H 10% extract (G4), (e) SFM-CM-NP-H 5% extract (G5), (f) SFM-CM-NP-H 10% extract (G6), (g) positive control (cells in DMEM-KO + 10% FBS) (G7), and (h) negative control (cells in DMEM-KO).

The metabolic activity of the cells was compared using an MTT assay, 24 and 48 h after treatment with the extracts. At the specific time points, 30 μL of MTT reagent (5 mg/mL in DPBS) was added to each well. The plates were incubated in the dark at 37 °C for 2 h, till the formation of visible purple precipitate. Then the media containing MTT was slowly aspirated out and 100 μL of DMSO was added to dissolve the formazan crystals. After 15 min of incubation, the absorbance was read at 570 nm with a reference filter of 620 nm, using a SpectraMax M3 microplate reader. The metabolic activity of the cells was expressed in terms of relative cell viability (%) normalized with respect to the negative control (NC), using the following formula:

$$ \mathrm{Relative}\ \mathrm{cell}\ \mathrm{viability}\ \left(\%\right)=\left(\mathrm{OD}\ \mathrm{of}\ \mathrm{sample}/\mathrm{OD}\ \mathrm{of}\ \mathrm{NC}\right)\times 100 $$

### Statistical analysis

The data is presented as mean ± standard deviation (SD). All graphs were plotted and statistical analysis carried out in GraphPad Prism (version 5.01). Statistical differences were analyzed wherever applicable using the following tests. Normal distribution was confirmed by using the D’Agostino and Pearson omnibus normality test and Kolmogorov-Smirnov test. Equal variance was determined by Bartlett’s test and *F* test when assuming Gaussian distribution. For multiple comparisons among groups one-way analysis of variance (ANOVA) followed by with Tukey’s multiple comparison test were performed for MSC cell count, viability, average diameter, circularity, and hydrogel porosity. Two-way ANOVA with Bonferroni post hoc tests for protein release from NPs, swelling kinetics, degradation rate, protein release from hydrogels, direct cell seeding on hydrogels, and cell treatment with hydrogel extracts. In order to compare between two groups, unpaired two-tailed column *t* tests were performed for protein content in CM and L-CM, NP diameter, zeta potential, and protein loading capacity. The level of statistical significance was set to *p* < 0.05.

## Results

### MSC characterization

**Fig. 1:**
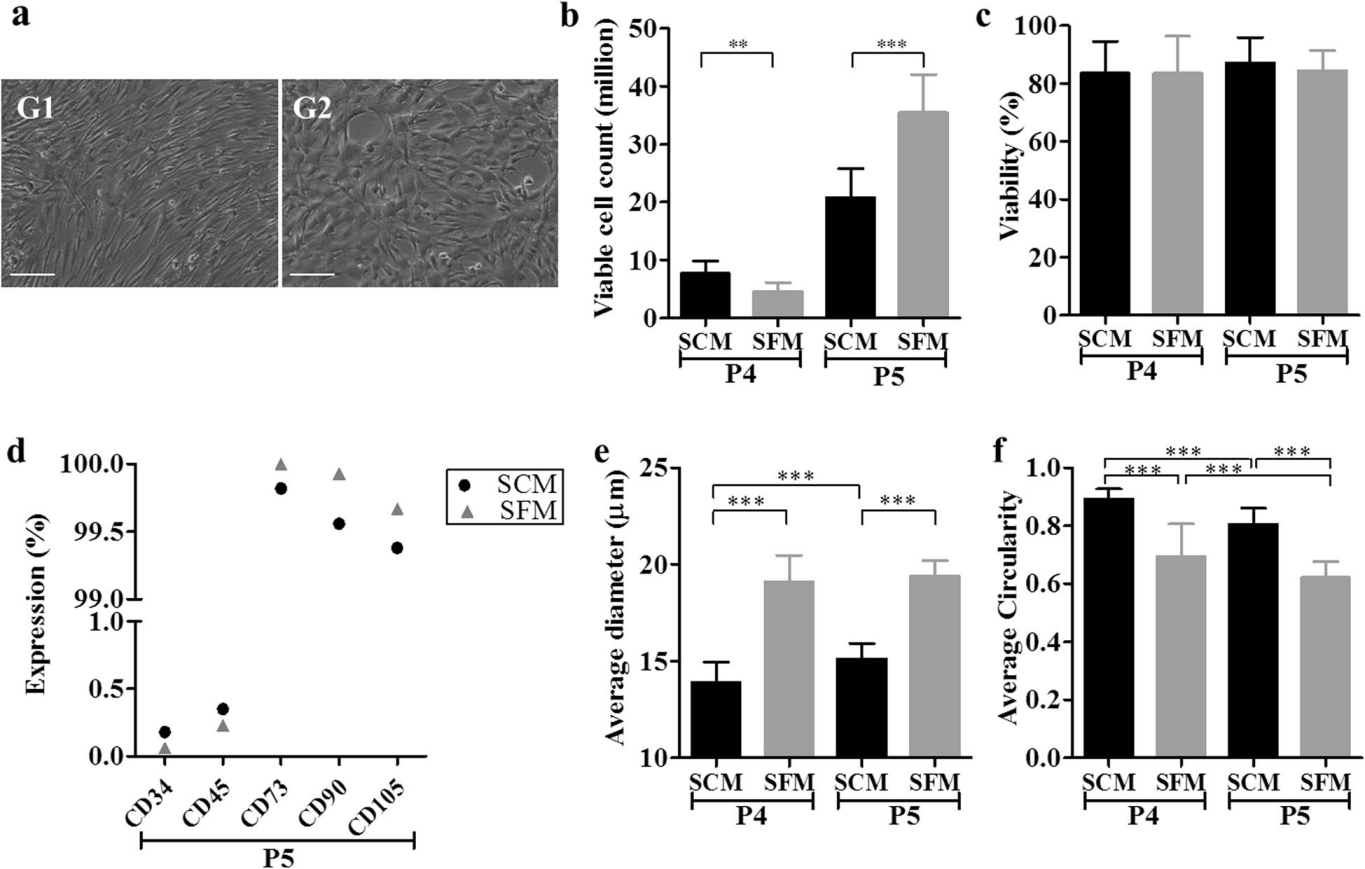
Characterization of MSCs cultured in SCM and SFM. **a** Morphology of P5 MSCs cultured in SCM (G1) and SFM (G2), **b** viable cell count, **c** cellular viability, **d** surface marker expression determined by flow cytometry, **e** average diameter, and **f** average circularity. (***p < 0.001, **p < 0.01,*p < 0.05). Abbreviations: SCM, serum-containing medium; SFM, serum-free medium

Cryopreserved P3 BM-MSCs were thawed and seeded onto culture flasks using SCM and SFM at a density of 1000 cells/cm^2^. The MSCs that were cultured using SCM and SFM exhibited spindle-shaped morphology as shown in Fig. 1a. MSCs cultured in SFM exhibited a larger size in comparison with MSCs in SCM.

Viable cell count was significantly higher in SCM cultured MSCs compared to SFM-cultured MSCs at P4 (**p < 0.01) (Fig. 1b). However, at P5, the viable cell count was significantly higher in SFM-cultured MSCs compared to SCM-cultured MSCs (***p < 0.001) (Fig. 1b). The percentage viability of cells cultured in SCM and SFM was above 80% and there was no significant difference observed between the groups (Fig. 1c).

The average diameter of the cells cultured in SCM significantly increased from P4 to P5 (***p < 0.001) while there were no significant differences observed between P4 and P5 of cells cultured in SFM. Interestingly, cells cultured in SFM were shown to be significantly higher in diameter both at P4 and P5 compared to MSCs cultured in SCM (***p < 0.001) (Fig. 1e). The average circularity of cells was measured between 0 and 1, where 1 indicates close to round shape and 0 indicates non-circular objects. The average circularity of MSCs cultured in SCM was significantly higher compared to MSCs cultured in SFM at P4 (***p < 0.001) and P5 (***p < 0.001) (Fig. 1f). Results indicate that the MSCs cultured in SCM are close to circular after harvest while MSCs cultured in SFM are non-circular.

**Fig. 2:**
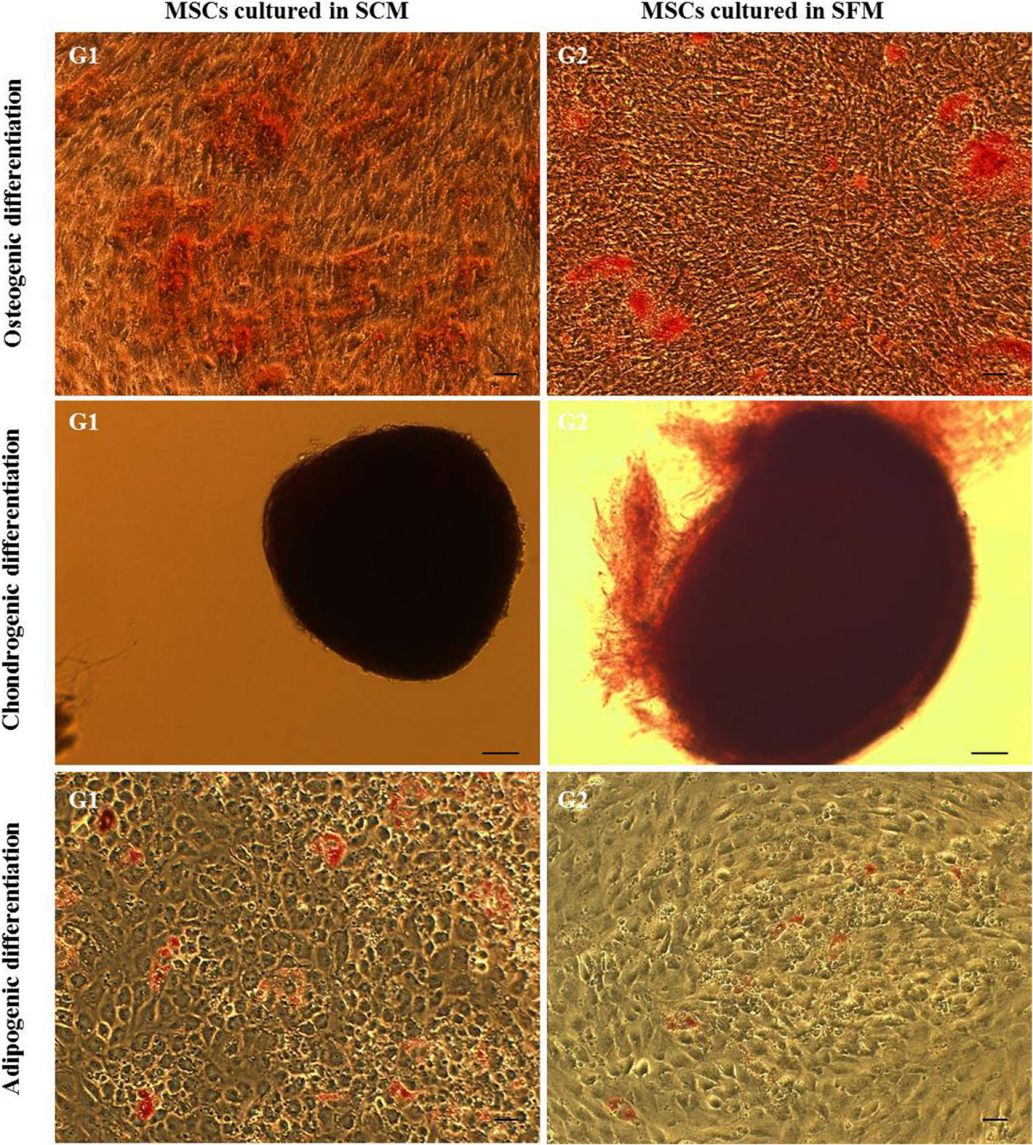
Trilineage differentiation of MSCs cultured in SCM (G1) and SFM (G2) conditions. Osteogenic differentiation evidenced by von Kossa’s staining, chondrogenic differentiation evidenced by Safranin-O staining, and adipogenic differentiation evidenced by Oil Red O staining. Abbreviations: SCM, serum-containing medium; SFM, serum-free medium

The expression of CD34 and CD45 was less than 1%, and CD73, CD90, and CD105 were above 99% in cells cultured with SCM and SFM (Fig. 1d). According to the ISCT criteria, MSCs are defined by their trilineage differentiation capacity. This was tested by analyzing osteo-, adipo-, and chondrogenic differentiation potential of the MSCs cultured in SCM and SFM. Typical morphological changes related to differentiation were observed for both sets of MSCs. Osteogenic differentiation was characterized by mineral deposits in the wells for both MSCs cultured in SCM (G1) and SFM (G2) (Fig. 2). Intracellular oil droplet formation was visible in adipogenic differentiation (Fig. 2) while chondrogenic mass formation was observed in the chondro-induced MSCs cultured in SCM (G1) and SFM (G2) (Fig. 2). The results indicate that MSCs cultured using SCM and SFM are capable of trilineage differentiation. The MSCs obtained by both culture conditions were compliant with the ISCT guidelines with respect to their morphology and CD surface marker expression and trilineage differentiation.

### CM characterization

The CM and L-CM were analyzed for total protein content using the Bradford assay and results are shown in Table 1. The quantified protein (mg/mL) for CM was normalized with the total number of cells obtained during the P5 culture. The ScCM significantly had a higher quantity of total protein compared to SfCM (***p < 0.001). Consistent with CM total protein content, lyophilized protein concentrations were significantly higher in ScCM (L-ScCM) compared to SfCM (L-SfCM) (***p < 0.001) (Table 1). In addition, the level of potency markers such as VEGF and TGF-β were significantly higher in ScCM compared to SfCM (***p < 0.001) (Table 2). BSA content in SfCM was quantified, as a potential xeno marker, and the level of BSA obtained was only in nanograms.

**Table 1:** Total protein content in conditioned medium (*n* = 2) and lyophilized conditioned medium (*n* = 3)

Sample	Quantified protein (mean ± SD)
ScCM	0.210 ± 0.006 mg/mL/million
SfCM	0.096 ± 0.011 mg/mL/million
L-ScCM	59.151 ± 13.888 μg/mg of L-CM
L-SfCM	39.849 ± 14.587 μg/mg of L-CM

Abbreviations: *ScCM* serum-containing conditioned medium, *SfCM* serum-free conditioned medium, *L-ScCM* lyophilized serum-containing conditioned medium, *L-SfCM* lyophilized serum-free conditioned medium

**Table 2:** Potency and xeno marker characterization in conditioned medium (*n* = 2)

Sample	Marker	Quantity (mean ± SD)
ScCM	VEGF	0.105 ± 0.043 ng/mL/million cells
SfCM		0.087 ± 0.009 ng/mL/million cells
ScCM	TGF-β	0.09 ± 0.005 ng/mL/million cells
SfCM		0.021 ± 0.001 ng/mL/million cells
SfCM	BSA	2.58 ng/mL

Abbreviations: *ScCM* serum-containing conditioned medium, *SfCM* serum-free conditioned medium, *VEGF* vascular endothelial growth factor, *TGF-β* transforming growth factor-β, *BSA* bovine serum albumin

### NP characterization

**Fig. 3:**
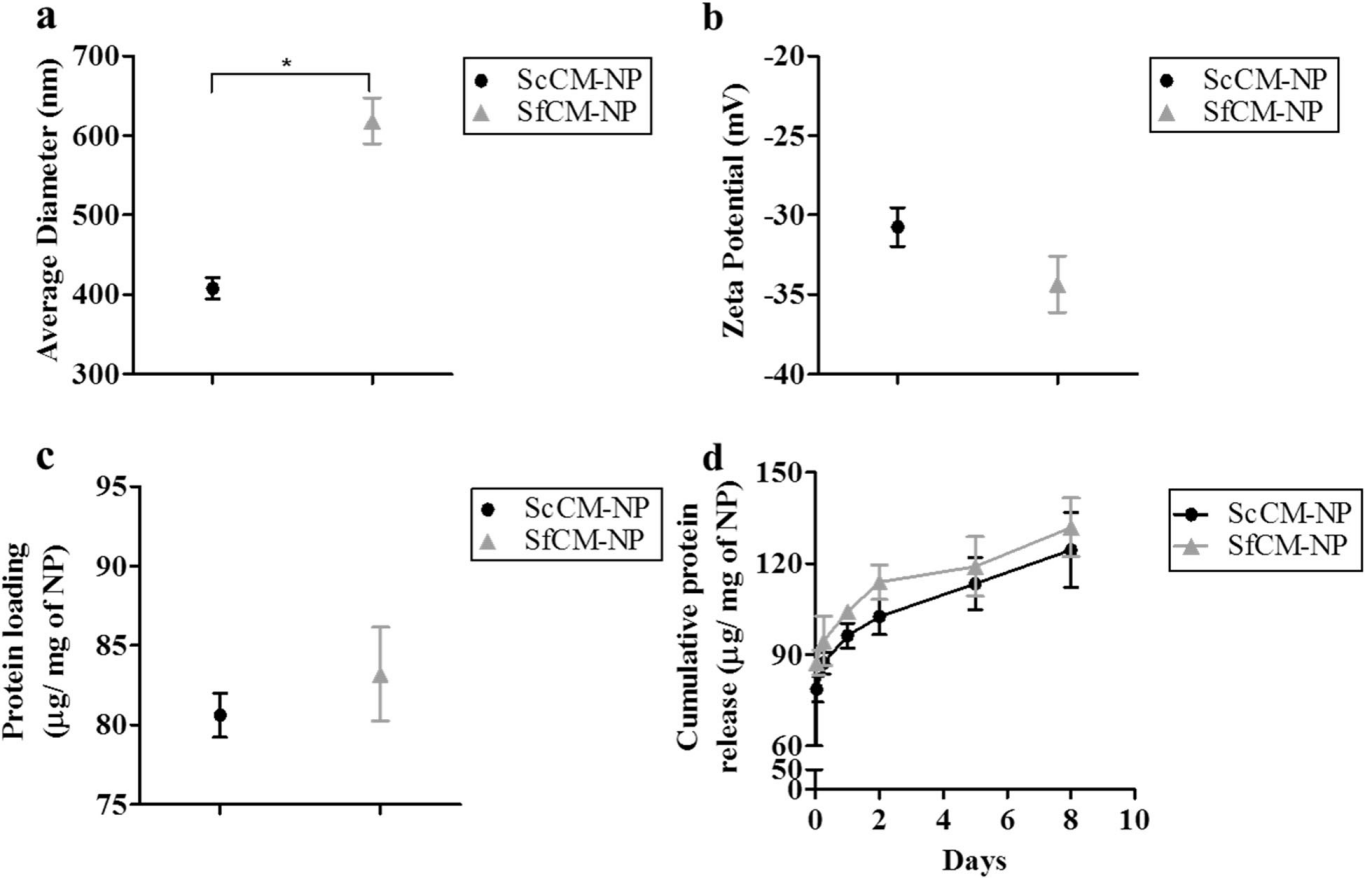
Characterization of nanoparticles encapsulated with MSC-CM. **a** Average diameter, **b** zeta potential, **c** protein loading content, and **d** protein release. Abbreviations: MSC-CM, MSC conditioned medium; ScCM-NP, serum-containing conditioned medium nanoparticle; SfCM-NP, serum-free conditioned medium nanoparticles

The average diameter and zeta potential of ScCM-NPs and SfCM-NPs were analyzed by dynamic light scattering (DLS). We found the average diameter of NP dispersed in aqueous dispersion was 407.95 nm for ScCM-NPs and 618.55 nm for SfCM-NPs (Fig. 3a). The ScCM-NPs exhibited a zeta potential of − 30.75 mV and SfCM-NPs of − 34.35 mV (Fig. 3b).

Further, protein content was extracted from NP using 0.1 M NaOH and 5% SDS to determine the protein loading capacity. Quantified protein from ScCM and SfCM-NPs was 80.6 and 83.2 μg/mg of NPs, respectively (Fig. 3c). Extraction was also carried out using 0.1 M NaOH + 1% SDS and results were comparable to the above data (not shown).

Furthermore, protein release patterns were similar for both groups of NPs with an initial burst release followed by a steady increase (Fig. 3d). At 1 h, protein release for ScCM-NPs and SfCM-NPs was 78.74 ± 4.29 μg/mg of NP and 87.42 ± 3.98 μg/mg of NP, respectively. Protein release at day 8 was 124.5 ± 12.3 μg/mg of NP and 131.9 ± 9.5 μg/mg of NP, respectively. There were no significant differences between the groups at any time points (Fig. 3d).

### Hydrogel-NP composite characterization

**Fig. 4:**
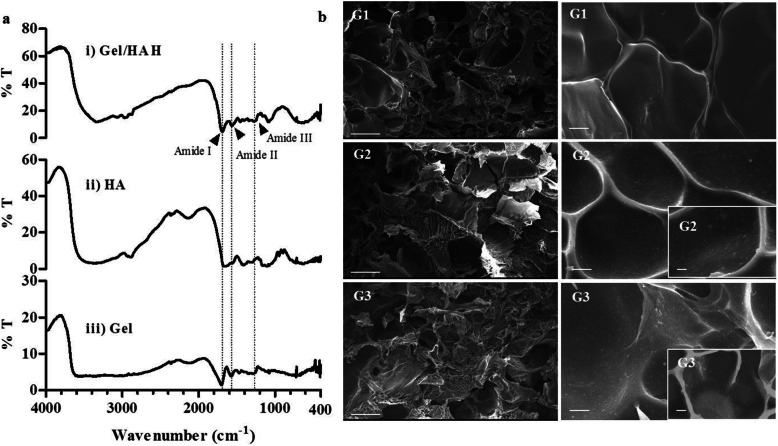
Chemical characterization of hydrogel and morphology of nanoparticle-hydrogel composite. **a** Fourier transform infrared (FTIR) spectra of gelatin (Gel), hyaluronic acid (HA), and gelatin-hyaluronic acid (Gel/HA) hydrogel composite. The characteristic peaks in the Gel/HA hydrogel spectrum centered at 1690, 1564, and 1279 assigned as amide bands I, II, and III. **b** Scanning electron microscope (SEM) images of the surface of Gel/HA hydrogel composite (G1), ScCM-NP-H composite (G2), and SfCM-NP-H composite (G3). Scale: G1, G2, G3 (left), 500 μm; G1, G2, G3 (right), 10 μm (× 1000 magnification); and G2, G3 (lower right corner), 10 μm (× 2000 magnification). Abbreviations: ScCM-NP-H, serum-containing conditioned medium encapsulated in nanoparticles—embedded in hydrogel composite; SfCM-NP-H, serum-free conditioned medium encapsulated in nanoparticles—embedded in hydrogel composite

Gelatin (Gel), hyaluronic acid (HA), and Gel/HA hydrogel were characterized using Fourier transform infrared spectroscopy (FTIR). The characteristic peaks in the Gel/HA hydrogel spectrum centered at 1690, 1564, and 1279 could be assigned to amide bands I, II, and III. These peaks exhibit higher transmittance in Gel/HA hydrogel compared to Gel and HA (Fig. 4a). Scanning electron microscopy (SEM) revealed the Gel/HA hydrogel consisted of a smooth textured surface (Fig. 4b, G1) while ScCM-NP-H and SfCM-NP-H composite hydrogels exhibited uniform distribution of NPs across the surface (Fig. 4b, G2 and G3).

**Fig. 5:**
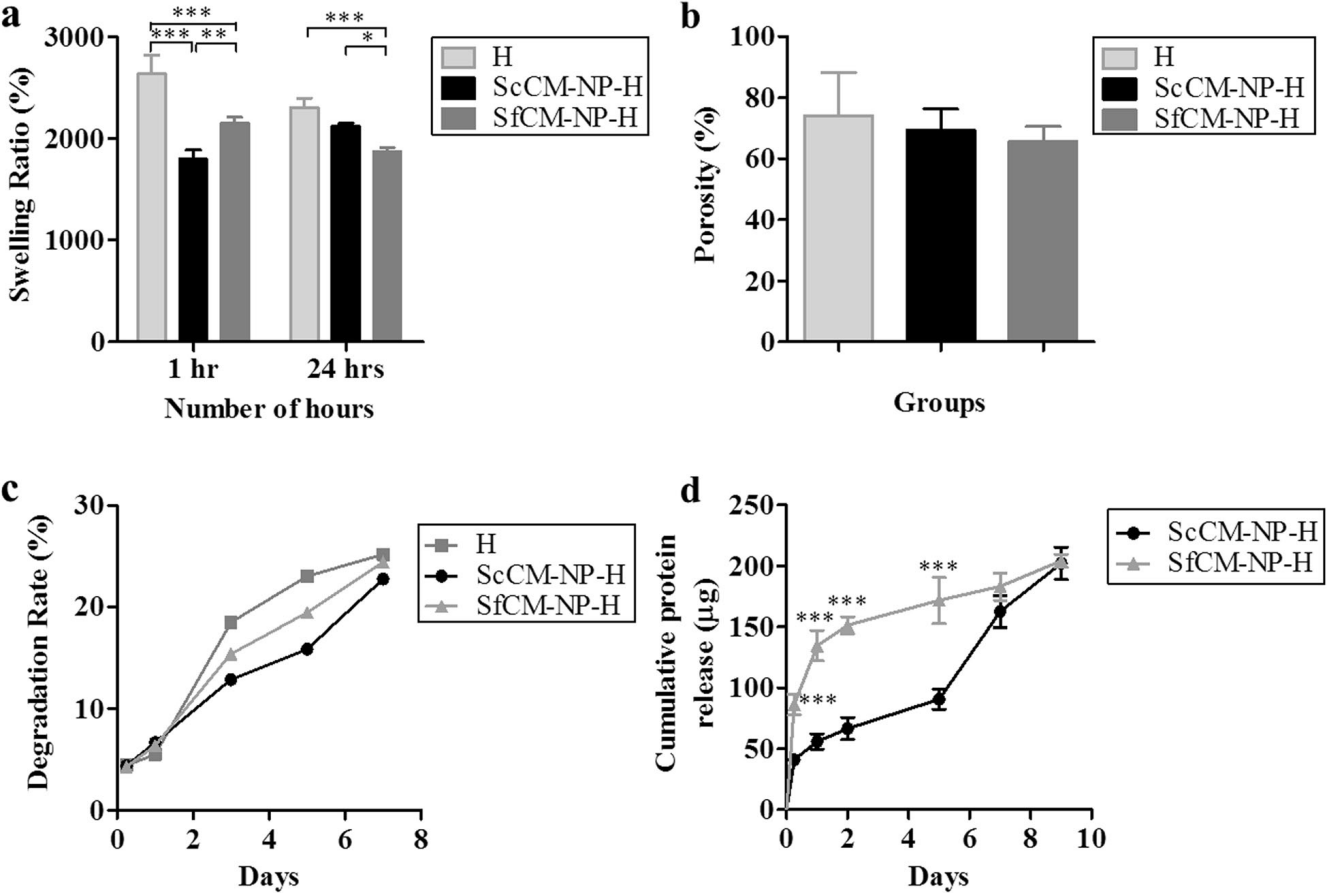
Characterization of the hydrogel composites. **a** Swelling kinetics, **b** porosity, **c** degradation rate, and **d** cumulative protein release (*n* = 3, ***p < 0.001, **p < 0.01, *p < 0.05). Abbreviations: H, hydrogel composite alone; ScCM-NP-H, serum-containing conditioned medium encapsulated in nanoparticles—embedded in hydrogel composite; SfCM-NP-H, serum-free conditioned medium encapsulated in nanoparticles—embedded in hydrogel composite

Further, hydrogel NP composites were characterized for porosity using a liquid displacement method, which confirmed that all groups have a porosity higher than 60%, with no significant differences between the groups (Fig. 5a). When the swelling kinetics were tested at 1 and 24 h, the swelling ratio of Gel/HA (H) after 1 h was 2640%, which was significantly reduced to 2308% at 24 h. The swelling ratio of ScCM-NP-H increased from 1799% at 1 h to 2124 at 24 h. On the other hand, the swelling ratio for SfCM-NP-H decreased from 2154% at 1 h to 1877% at 24 h (Fig. 5b). Furthermore, the degradation pattern at days 3 and 5 was slower in ScCM-NP-H compared to the other groups, but this was not statistically significant. At the end of 7 days, the difference was reduced: 22.74% in ScCM-NP-H, 24.4% in SfCM-NP-H, and 25.17% in H (Gel/HA) (Fig. 5c).

We further elucidated the protein release pattern from the NP-H composite groups. SfCM-NP-H showed an initial burst release for 2 days, followed by a sustained release. In ScCM-NP-H, the release was sustained over the course of 5 days, after which a surge was observed. The release in SfCM-NP-H was significantly higher than ScCM-NP-H at all time points up to day 5. By the end of 9 days, the release rate became comparable for both groups (Fig. 5d).

### Functional evaluation of hydrogel NP composite

**Fig. 6:**
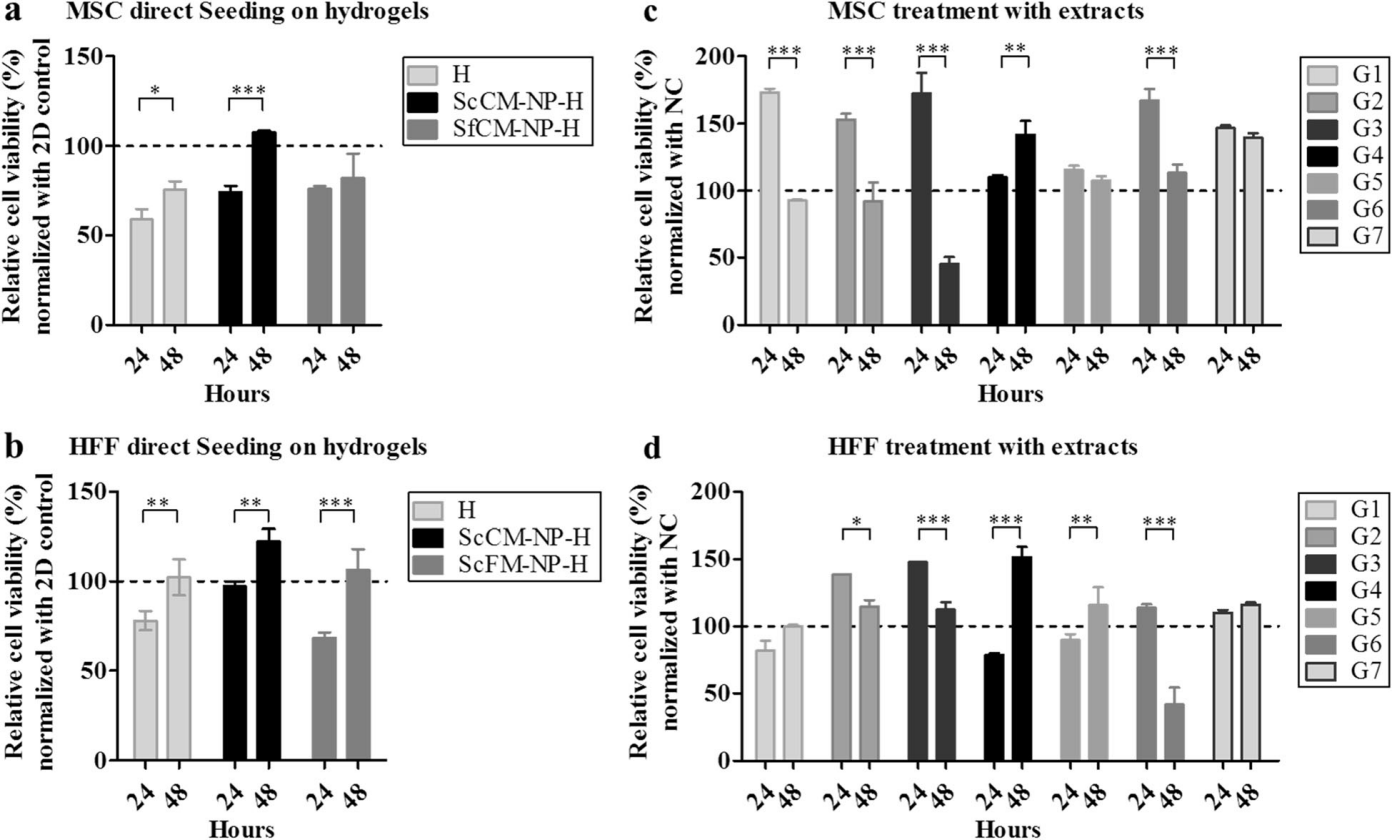
Functional evaluation of hydrogel composites loaded with nanoparticles containing conditioned medium (MTT assay). **a** Relative cell viability (%) of MSCs seeded on hydrogel composite (normalized with 2D control), **b** relative cell viability (%) of HFFs seeded on hydrogel composite (normalized with 2D control), **c** relative cell viability (%) of MSCs treated with extracts (normalized with NC), and **d** relative cell viability (%) of HFFs treated with extracts (normalized with NC) (***p < 0.001, **p < 0.01, *p < 0.05). Abbreviations: ScCM-NP-H, serum-containing conditioned medium encapsulated in nanoparticles—embedded in hydrogel composite; SfCM-NP-H, serum-free conditioned medium encapsulated in nanoparticles—embedded in hydrogel composite. G1: 5% H extract; G2: 10% H extract; G3: 5% ScCM-NP-H extract; G4: 10% ScCM-NP-H extract; G5: 5% SfCM-NP-H extract; G6: 10% SfCM-NP-H extract; G7: DMEM+ 10% FBS

We next tested the potential cytotoxicity and biocompatibility of the hydrogel-NP composite in MSCs and human fibroblasts (HFFs). When MSCs were seeded onto the hydrogel composite and stained with MTT at 24 h and 48 h, we found that the metabolic activity increased in a time-dependent manner. At 24 h, the relative metabolic activities of MSCs seeded on H, ScCM-NP-H, and SfCM-NP-H were 58.7, 74, and 75.9%, respectively. At 48 h, there was a significant increase in the metabolic activity for H and ScCM-NP-H with 75.4% (*p < 0.05) and 107.5% (***p < 0.001), respectively, while MSCs on SfCM-NP-H, maintained their activity at 82.1% (Fig. 6a). Similarly, at 24 h, the relative metabolic activities of HFFs seeded on H, ScCM-NP-H, and SfCM-NP-H were 78, 98.2, and 68.3%, respectively. After 48 h, the relative metabolic activity in H, ScCM-NP-H, and SfCM-NP-H increased significantly to 102.1 (*p* < 0.01), 122.3 (*p* < 0.01), and 106.4% (*p* < 0.001), respectively (Fig. 6b).

We further evaluated the relative metabolic activities of MSCs and HFFs treated with different concentrations of hydrogel extracts at 24 and 48 h. Initially, MSCs were treated with 50% extracts, but this presented low absorbance readings (data not shown). In fact, the cells were unhealthy and showed no proliferation traits. Therefore, the cells were treated with 5% and 10% concentrations of extracts. The metabolic activity of MSCs (Fig. 6c) treated with 5% H extract (G1), 10% H extract (G2), 5% ScCM-NP-H extract (G3), and 10% SfCM-NP-H (G6) extract showed duration-dependent decreases in activity from 173.3, 153.2, 172.3, and 166.9% at 24 h to 92.6 (***p < 0.001), 92.2 (***p < 0.001), 45.2 (***p < 0.001), and 113.1% (***p < 0.001), respectively. In contrast, the metabolic activity of 10% ScCM-NP-H (G4) showed duration-dependent increases in activity from 109.9% at 24 h to 141.6% (**p < 0.01) at 48 h. On the other hand, metabolic activity of 5% SfCM-NP-H (G5) did not show significant differences at 24 and 48 h.

Further, when we analyzed the metabolic activity of HFF (Fig. 6d) treated with 10% H extract (G2), 5% ScCM-NP-H extract (G3), and 10% SfCM-NP-H extract (G6), we found they were significantly reduced from 138.5, 147.7, and 113.7% at 24 h to 114.5 (**p < 0.01), 112.3 (***p < 0.001), and 41.7% (***p < 0.001), respectively, at 48 h. Consistent with MSC metabolic activity results, HFFs treated with 10% ScCM-NP-H extract (G4) showed increases in activity from 78.5% at 24 h to 151.4% (***p < 0.001) at 48 h. In addition, the metabolic activity of HFF treated with 5% SfCM-NP-H extract (G5) was increased significantly from 89.7% at 24 h to 115.6% (**p < 0.01) at 48 h. On the other hand, 5% H extract (G1) did not show significant differences in metabolic activity at 24 or 48 h.

## Discussion

MSC-CM has immense potential in the field of regenerative medicine and its therapeutic potency has been established during the past few years, although mainly in animal models, thus its translation into the clinic settings is still in the early stages. SCM is commonly used for MSC culture, but the presence of xenogeneic compounds is its major drawback [18, 20]. In this study, we have compared the differences between MSCs cultured using SCM and a commercially available SFM. MSCs were adherent and exhibited spindle-shaped morphology, though MSCs cultured in SFM were larger in size, with respect to morphology and average diameter. MSC circularity decreased with increasing passage number in SCM culture, and SFM-cultured MSCs had the lowest circularity. These features correlate with the heterogeneity associated with BM-MSCs when cultured in serum [8] and it is a microenvironment-dependent response. Viability of the cells was comparable, while viable cell count was significantly higher for SFM-cultured MSCs. Both SCM- and SFM-cultured MSCs were compliant with the ISCT guidelines, in terms of morphology and surface marker expression and trilineage differentiation capacity under in vitro culture conditions [4]. MSCs were cultured through P4 and P5 in SCM and SFM, following which the respective CM was collected. ScCM and SfCM were then characterized for total protein content. Although cell yield was higher in SFM-cultured MSCs, ScCM showed significantly higher total protein content. Nevertheless, it is difficult to differentiate whether this is due to increased protein secretion by MSCs in SCM or due to the presence of serum proteins that were not completely utilized by the cells during the culture period. VEGF and TGF-β are two of the most important factors involved in the tissue repair and regeneration process; therefore, the potency of CM was estimated by quantification of these factors [34, 35]. VEGF and TGF-β secretions were found to be comparable for ScCM and SfCM. Also, BSA content in SfCM was found to be minimal, which was the main purpose of utilizing SFM.

Bioactive factors present in the MSC-CM are available in highly diluted conditions in the culture medium. The concentration of CM ensures greater bioavailability of these factors for therapeutic applications. Lyophilization removes the liquid component, thereby acquiring a highly concentrated form of the CM, which also has a greater shelf-life and can easily be reconstituted in the desired media [12]. Peng et al. fabricated a freeze-dried rat BM-MSC-CM membrane that was able to retain 80% of paracrine factors upon rehydration, and also demonstrated enhanced fibroblast survival and wound healing in rat wound models [36]. We used 4% trehalose during lyophilization of the CM to protect against any freezing/dehydration stresses. Total protein content in L-ScCM was 59.15 ± 13.8 μg/mg of L-CM, and this is comparable to the results obtained by Bari et al. [29] for lyophilized adipose-derived MSC-CM.

Polymer NPs have been used extensively for protein encapsulation, but suffer due to their high burst release [30]. The polymer matrix usually degrades by erosion, resulting in the formation of pores which allow even high molecular weight proteins to be released in a controlled manner [37]. The double emulsion solvent evaporation method used here yielded CM-NPs with comparable properties and protein release pattern showed an initial burst release followed by steady release. SfCM-NPs were larger in diameter; however, the zeta potential and protein release profiles were identical for both groups. Although total protein content in L-ScCM was higher than L-SfCM, this factor was not reflected during NP characterization.

Hydrogels facilitate protein delivery by a diffusion-controlled mechanism resulting from the swelling of the polymer matrix. Incorporation of NPs into hydrogels can modulate the release kinetics in ways that the individual components cannot achieve [22]. Here, we compared a Gel/HA hydrogel (H) with ScCM-NP-H and SfCM-NP-H. Swelling ratios were higher than 1500% for all groups, indicating a high water retention capacity. Porosity was comparable in all groups and the values correlate with that of swelling kinetics. Degradation rates indicated 20–25% weight loss during the analysis period. Protein release rate is linked to swelling ratio, porosity, and degradation rate. We have observed that there is slightly higher porosity and lower degradation rate in ScCM-NP-H compared to SfCM-NP-H, though statistically insignificant. Protein release rate in ScCM-NP-H is higher until day 6 compared to SfCM-NP-H even though slightly higher swelling ratio and porosity and lower degradation rate of ScCM-NP-H compared to SfCM-NP-H. In addition, it is essential to note that the presence of serum protein may increase encapsulation stability and controlled release of secretome components. As gelatin is also a protein, it can possibly interfere with the total quantified protein in the release study. Thus, based on the structural characterization and protein release rate, the hydrogel composite can be successfully prepared with a controlled release of MSC-CM components. However, the controlled release pattern of proteins needs to be considered for further elucidation. Also, it is essential to attribute the highest metabolic activity of ScCM-NP-H to the unused serum contents or different SCM cultured MSC secretome contents.

In many instances, support for recruitment, growth, and differentiation of tissue-specific progenitor cells is essential. In addition, fibroblasts play an essential role during the tissue repair stages of cell replication and matrix synthesis by secreting extracellular matrix components thereby maintaining complex interactive microenvironment. Human MSCs and fibroblasts used in this study possibly mimic some aspects observed under physiological conditions in vivo. In this context, we have evaluated the CM-NP-H composite biocompatibility and metabolism using MSCs and fibroblasts. MSCs and HFFs seeded onto the NP-H composites displayed comparable metabolic activities with the control groups, indicating the biocompatible nature of the NP-H composites. The major trend in the MSCs and HFFs treated with 5% and 10% NP-H extract groups was an increased metabolic activity at 24 h followed by a rapid decline at 48 h. The main exception was 10% SCM-NP-H extract, which increased the metabolic activities of MSCs as well as HFFs after 48 h of treatment. This variation between 5 and 10% extracts indicates that the bioactive factors present in the 10% ScCM-NP-H extract are capable of maintaining cellular activity till 48 h. The metabolic activity of seeded cells showed a significant rise from 24 to 48 h, indicative of sustained release of bioactive factors from all the NP-H composite groups. Here, the protein release pattern can be correlated with relative cell viability result of MSCs. Slow release of secretome components from ScCM-NP-H has significantly influenced duration-dependent metabolic activity of MSCs compared to SfCM-NP-H groups. This effect was more profound in HFFs despite the varying levels of release rate from ScCM-NP-H and SfCM-NP-H groups displaying metabolic activities greater than the 2D control by the end of 48 h. It should be noted that the concentration of bioactive factor secretions in the picogram or nanogram range was sufficient to induce significant cellular responses [11, 38, 39] and synergistic effects [40, 41]. Furthermore, direct treatment of the hydrogel-NP extract exhibited a strong anti-metabolic effect on MSC and HFF thus signifying the potential scope of ScCM-NP-H composites for sustained release of bioactive factors.

In this study, we have utilized hyaluron, gelatin, and PLA to prepare the CM-NP-hydrogel composite. It should be pointed out that our work represents the first example of utilizing a NP-hydrogel composite to deliver MSC-CM in a controlled manner. Thus, we have successfully demonstrated the biocompatibility and strong metabolic effect of CM-NP-hydrogel composites. MSC-derived CM is known to mediate wound healing and alleviate osteoarthritis in animal models. A proposed model of MSC-CM-NP-hydrogel can be used as an injectable for wound healing and cartilage regeneration applications. This model can enable numerous applications in the development of biomedical nanodevices and various tissue engineering applications such as skin and corneal wound healing, neuroprotection and spinal cord repair, and cartilage repair. Further, Gel-HA hydrogel properties can be modified to alter physical properties and controlling the delivery of MSC-CM either as injectable or as an implant material for soft and hard tissue repair and regeneration.

## Conclusion

To utilize the immense potential of MSC-CM, we have developed an NP-hydrogel composite and characterized the physical and structural features of NP, the hydrogel, and its composite. We have shown that MSC-CM can be encapsulated into nanoparticles and that can be integrated into a hydrogel to form a composite without losing MSC-CM functional properties. The MSC-CM-NP-hydrogel composite exhibited biocompatibility by supporting HFF and MSC adhesion and proliferation. Furthermore, controlled release of MSC-CM from the NP-hydrogel composite promoted the metabolic activity in HFF and MSC demonstrating the potential scope for regenerative medicine. Future work will examine the pores within the hydrogel, swelling ratio, cell infiltration and distribution, and tissue formation in the presence of conditioned medium under the right conditions. Given the shortcomings of CM-NP-H composite delivery system, this study has provided the basis for using the MSC-CM-based NP-hydrogel composite as a novel system for effective MSC-CM-based tissue engineering applications.

## Supplementary information

**Additional file 1: Fig. S1.** Morphology of MSCs seeded on hydrogel (H), ScCM-NP-H and SfCM-NP-H. Abbreviations: ScCM-NP: Serum containing conditioned medium nanoparticle; SfCM-NP: Serum free conditioned medium nanoparticles.

## Data Availability

All materials are available from the corresponding author.

## Abbreviations

BSA: Bovine serum albumin
DLS: Dynamic light scattering
FTIR: Fourier transform infrared
Gel/HA: Gelatin/hyaluronic acid
HFF-1: Human fibroblasts
ISCT: International Society for Cellular Therapy
L-ScCM: Lyophilized serum-containing conditioned medium
L-SfCM: Lyophilized serum-free conditioned medium
MSC-CM: Mesenchymal stromal cell conditioned medium
NP: Nanoparticles
NP-H: Nanocomposite-hydrogel
PLA: Poly-lactic acid
ScCM: Serum-containing conditioned medium
ScCM-NP: Serum-containing conditioned medium nanoparticle
ScCM-NP-H: Serum-containing conditioned medium encapsulated in nanoparticles—embedded in hydrogel composite
SCM: Serum-containing medium
SEM: Scanning electron microscope
SfCM: Serum-free conditioned medium
SfCM-NP: Serum-free conditioned medium nanoparticles
SfCM-NP-H: Serum-free conditioned medium encapsulated in nanoparticles—embedded in hydrogel composite
SFM: Serum-free medium
TGF-β: Transforming growth factor-β
VEGF: Vascular endothelial growth factor
