# Supplementary material for: A nanocomposite hydrogel delivery system for mesenchymal stromal cell secretome
Source: Stem Cell Res Ther. 2020 May 27;11:205. doi: 10.1186/s13287-020-01712-9 (PMC7251860; doi:10.1186/s13287-020-01712-9)
Supplement: Supplementary file 1 — Additional file 1: Fig. S1. Morphology of MSCs seeded on hydrogel (H), ScCM-NP-H and SfCM-NP-H. Abbreviations: ScCM-NP: Serum containing conditioned medium nanoparticle; SfCM-NP: Serum free conditioned medium nanoparticles. [file 13287_2020_1712_MOESM1_ESM.docx]

**Supplementary information**

**Method**

MSCs were seeded on the hydrogel (H) and conditioned medium (NP-H) composites and observed for 24 and 48 hours. Briefly, the H and NP-H composites were added to 48 well culture plates and washed twice with DPBS. The hydrogels were soaked in 200 μL of DMEM-KO for 24 hours. The saturated hydrogels were seeded with 2x10^5^ MSCs and incubated in a CO2 incubator. Morphology of the seeded MSCs was analysed using an inverted microscope at 24 and 48 hours. The groups were as follows: a) MSCs in H b) MSCs in ScCM-NP-H; c) MSCs in SfCM-NP-H.

**Results**

We have carried out a qualitative morphological evaluation of the MSCs under a microscope and analysed the penetration of the cells. MSCs seeded on the hydrogel (H) alone have retained round morphology with very few spindle-shaped cells observed at 24 hours. By 48 hours, both round and spindle shaped morphology have been observed in hydrogel alone. While the MSCs seeded on ScCM-NP-H showed spindle shaped morphology with very few round shaped cells at 24 and 48 hours. In comparison to ScCM-NP-H, cells seeded on SfCM-NP-H showed both round to spindle shaped morphology at 24 and 48 hours.


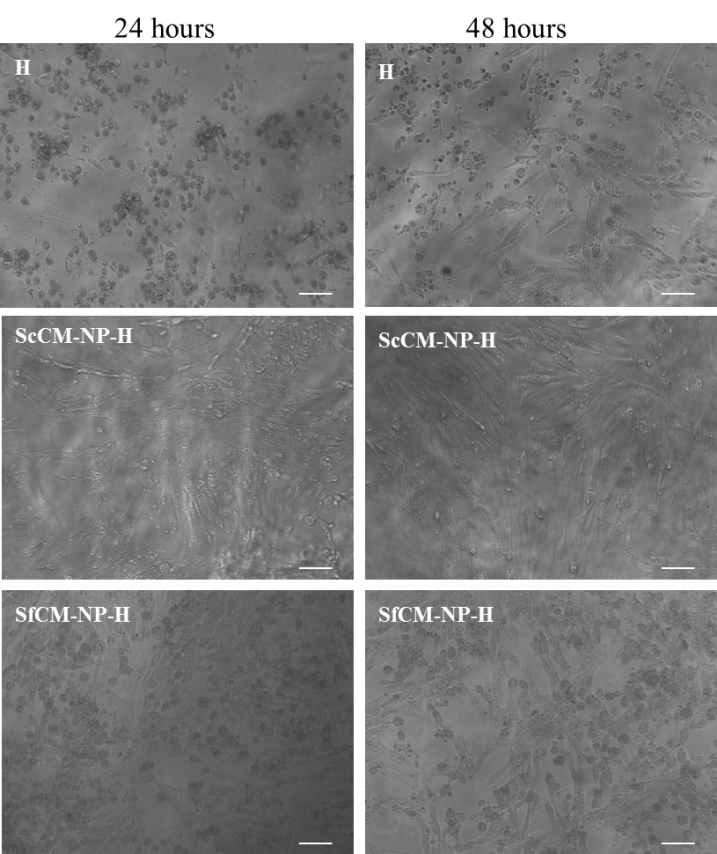


**Figure S1:** Morphology of MSCs seeded on hydrogel (H), ScCM-NP-H and SfCM-NP-H. Abbreviations: ScCM-NP: Serum containing conditioned medium nanoparticle; SfCM-NP: Serum free conditioned medium nanoparticles.

**Discussion and conclusion**

The MSCs appeared to attach to the hydrogel with spindle-shaped morphology (spread morphology). While the hydrogel alone supported the cell attachment and spreading, presence of CM components influenced cell attachment and formation of spindle shape. Interestingly, differences were observed between ScCM-NP-H and SfCM-NP-H with regard to spindle-shaped morphology formation in cells. MSCs were able to achieve high level of spindle shaped morphology on ScCM-NP-H compared to hydrogel alone and SfCM-NP-H. Further, these results are in correlation with biocompatibility and metabolic activity results of MSCs using MTT assay. Results of MTT assay showed significant increase in the relative metabolic activities of MSCs in H at 24 hours (58.7%) and 48 hours (75.4%, *p<0.05), in ScCM-NP-H at 24 hours (74%) and 48 hours (107.5%, *p<0.05) while ScCM-NP-H at 24 hours (75.9%) and 48 hours (82.1%) did not show significant increase. Although, CM components were present in ScCM-NP-H and SfCM-NP-H, it is possible that the unknown composition of serum in ScCM is able to promote cell spreading, allowing the cells to achieve increased functional activity. While this aspect of difference in SCM and SFM derived CM needs to be investigated further, it opens an exciting area to study pores within the hydrogel, swelling ratio, cell infiltration and distribution and tissue formation in the presence of conditioned medium.
